# Decreased TESK1-mediated cofilin 1 phosphorylation in the jejunum of IBS-D patients may explain increased female predisposition to epithelial dysfunction

**DOI:** 10.1038/s41598-018-20540-9

**Published:** 2018-02-02

**Authors:** Bruno K. Rodiño-Janeiro, Cristina Martínez, Marina Fortea, Beatriz Lobo, Marc Pigrau, Adoración Nieto, Ana María González-Castro, Eloísa Salvo-Romero, Danila Guagnozzi, Cristina Pardo-Camacho, Cristina Iribarren, Fernando Azpiroz, Carmen Alonso-Cotoner, Javier Santos, Maria Vicario

**Affiliations:** 1 Laboratory of Neuro-Immuno-Gastroenterology, Digestive System Research Unit, Vall d’Hebron Institut de Recerca, Department of Gastroenterology, Hospital Universitari Vall d’Hebron, Universitat Autònoma de Barcelona (Facultat de Medicina), Barcelona, Spain; 2 Translational Mucosal Immunology, Digestive System Research Unit, Vall d’Hebron Institut de Recerca; Department of Gastroenterology, Hospital Universitari Vall d’Hebron, Universitat Autònoma de Barcelona (Facultat de Medicina), Barcelona, Spain; 3 Centro de Investigación Biomédica en Red de Enfermedades Hepáticas y Digestivas (CIBERehd), Instituto de Salud Carlos III, Subdirección General de Investigación Sanitaria, Ministerio de Economía, Industria y Competitividad, Madrid, Spain

## Abstract

Disturbed intestinal epithelial barrier and mucosal micro-inflammation characterize irritable bowel syndrome (IBS). Despite intensive research demonstrating ovarian hormones modulation of IBS severity, there is still limited knowledge on the mechanisms underlying female predominance in this disorder. Our aim was to identify molecular pathways involved in epithelial barrier dysfunction and female predominance in diarrhea-predominant IBS (IBS-D) patients. Total RNA and protein were obtained from jejunal mucosal biopsies from healthy controls and IBS-D patients meeting the Rome III criteria. IBS severity was recorded based on validated questionnaires. Gene and protein expression profiles were obtained and data integrated to explore biological and molecular functions. Results were validated by western blot. Tight junction signaling, mitochondrial dysfunction, regulation of actin-based motility by Rho, and cytoskeleton signaling were differentially expressed in IBS-D. Decreased TESK1-dependent cofilin 1 phosphorylation (pCFL1) was confirmed in IBS-D, which negatively correlated with bowel movements only in female participants. In conclusion, deregulation of cytoskeleton dynamics through TESK1/CFL1 pathway underlies epithelial intestinal dysfunction in the small bowel mucosa of IBS-D, particularly in female patients. Further understanding of the mechanisms involving sex-mediated regulation of mucosal epithelial integrity may have significant preventive, diagnostic, and therapeutic implications for IBS.

## Introduction

In recent years, the conventional view of irritable bowel syndrome (IBS) as a non-organic disorder has been challenged by several reports showing the presence of neuronal damage, altered neuroplasticity, microbial dysbiosis and barrier dysfunction along with alterations in the expression and localization of intestinal epithelial tight junction (TJ) proteins^1^. Additionally, a low-grade infiltration of mast cells, lymphocytes and plasma cells and immune activation has been identified in the *lamina propria* of both large and small bowel mucosa, particularly in diarrhea-predominant IBS patients (IBS-D)^2–4^. Although, the true origin of the intestinal micro-inflammation and immune activation observed in IBS patients is still unknown, a potential pathophysiological link to these findings could be the alteration of the epithelial barrier function, that would lead to disturbed mucosal homeostasis^1,5–7^. In this line, we and others have recently shown altered expression, phosphorylation and localization of core TJ proteins, increased intercellular space and cytoskeleton condensation in jejunal and colonic epithelium of IBS^8^. These abnormalities may be partly mediated by mast cells through the activation of protease activated receptor 2 (PAR2), which can regulate epithelial barrier function, myosin light chain (MLC) phosphorylation and the expression of cytoskeleton proteins^9^. PAR2 is activated by serine proteases-mediated cleavage, and these, including tryptase and trypsin, have been shown to be enhanced in the jejunal fluid, feces and supernatants from colonic biopsies of IBS-D^10^. Moreover, PAR2 epithelial basolateral pools can regulate the actin depolymerization through cofilin 1 (CFL1) by beta-arrestin^11^. CFL1 has a leading role in actin depolymerization on the barbed end^12^ and can modulate epithelial permeability by TJ regulation^11,13,14^ and epithelial cell interactions with extracellular matrix^15^, events that could be related to cytoskeleton deregulation as observed in the jejunum of IBS-D^6,7^. Notably, phosphorylation of CFL1 by specific kinases LIMK and TESK1^16,17^ inactivates actin depolymerization which, in turn, is regulated by Rho GTPases and integrin/vinculin signaling, respectively^18^.

High throughput molecular techniques, such as microarray profiling and RNAseq, followed by accurate analysis of the associated biological pathways have provided new insights on the molecular mechanisms involved in the pathophysiology of IBS^6,19–23^. Proteomic tools and related strategies have been recently applied to profile protein expression in urine samples from IBS patients^24^ and in the colonic mucosa and the neuromuscular layer from rat models mimicking IBS clinical manifestations^25,26^. However, a comprehensive proteomic profiling in intestinal tissue from IBS patients has not been performed so far.

Our hypothesis was that alterations in molecular pathways modulating epithelial barrier dysfunction determine the female predominance observed in IBS-D patients. To test our hypothesis, we have applied an integrative multi-omics approach combining data from transcriptomic and proteomic profiling analysis in the jejunal mucosa of IBS-D patients *vs*. healthy controls further subdividing groups by sex.

## Results

### The jejunal mucosa of IBS-D shows a distinctive proteomic profile

**Figure 1:**
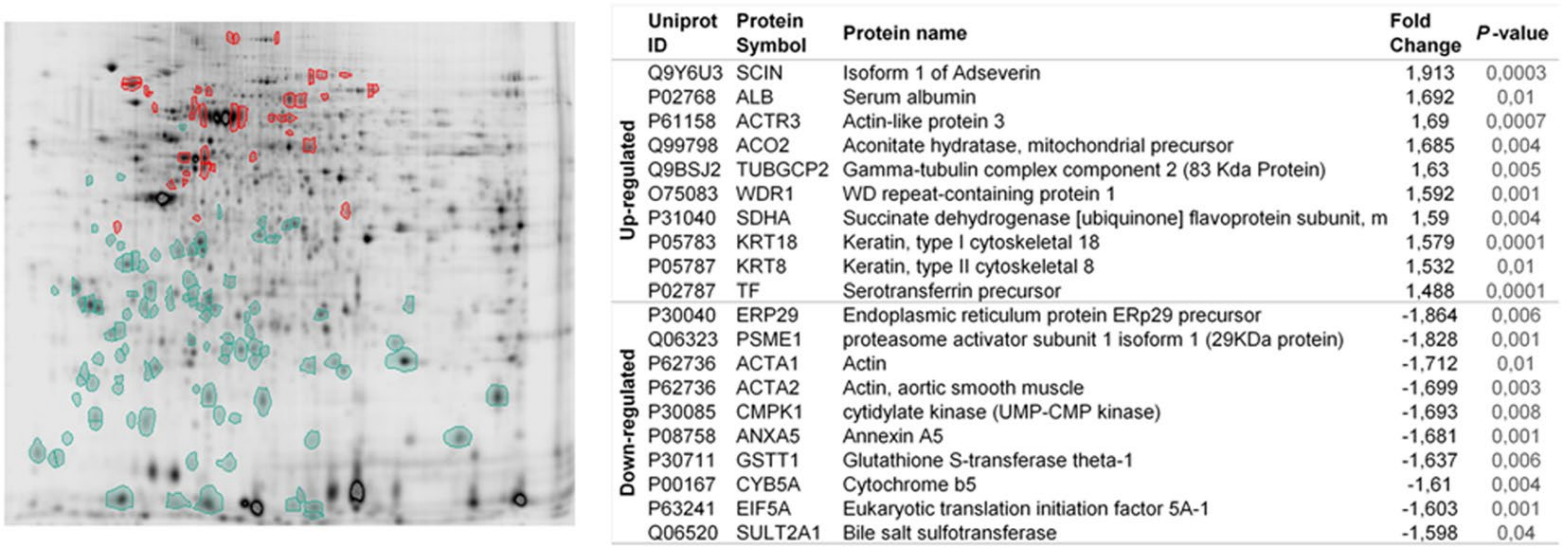
Proteomic signature of IBS-D patients. (**A**) DIGE analysis of protein extracts from jejunal mucosal biopsies. Heatmap for the 95 identified spots differentially expressed in IBS-D samples (n = 4) compared to healthy control samples (n = 4). Colors represent relative expression levels on a color scale (green: low/red: high). A total of 1200 spots were analyzed. 139 showed a >1.3-fold change and, from those, 95 spots were identified. Thirty-seven spots showed increased intensity (red-encircled spots), whereas 58 spots showed decreased intensity (green-encircled spots). (**B**) Annotations for the top ten up- and down-regulated spots identified in IBS-D *vs*. healthy control samples. Uniprot accession number, protein symbols and name, and their fold change expression in IBS-D with respect to healthy controls are shown.

**Figure 2:**
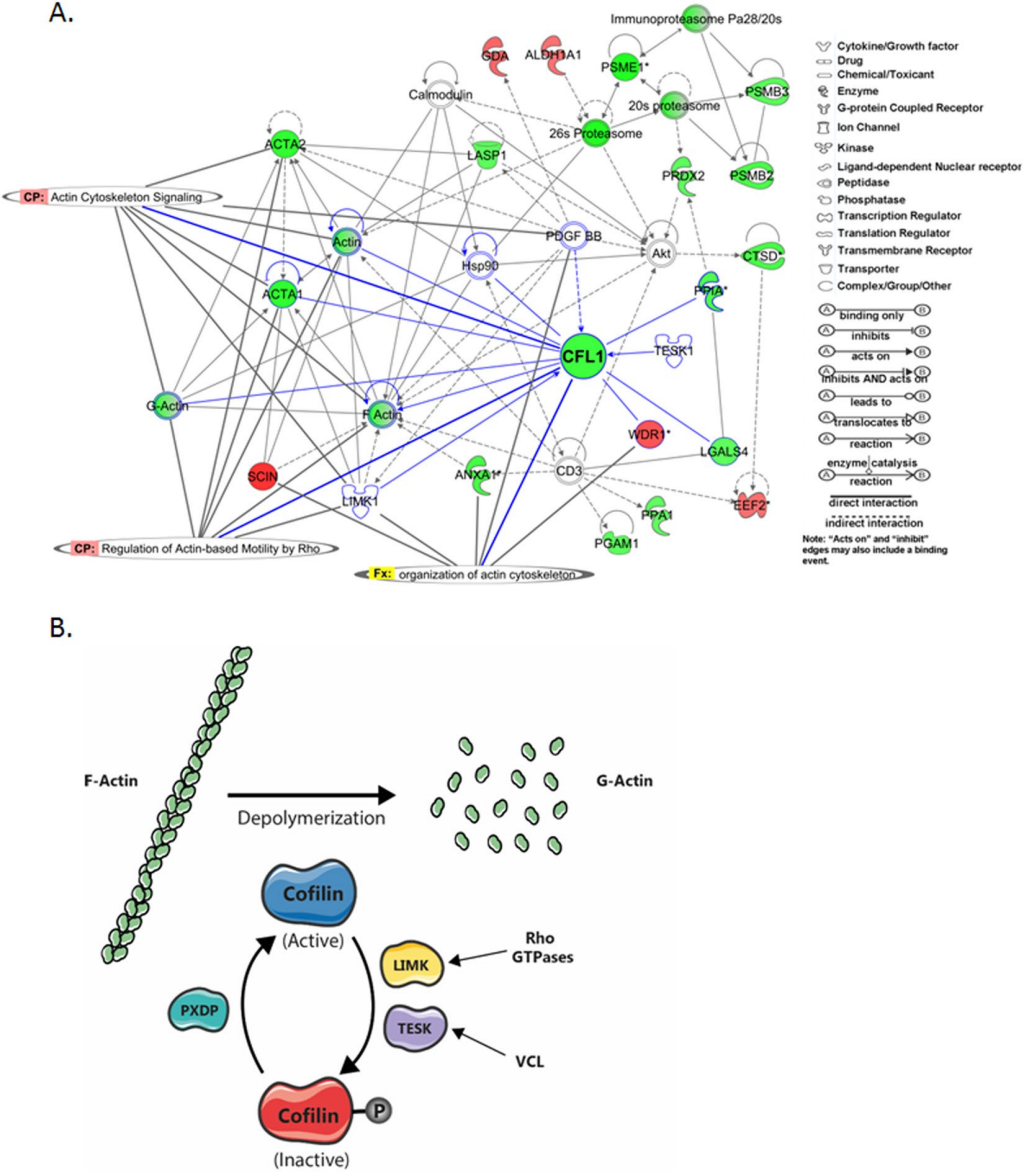
Relationships between differentially expressed proteins in the highest scored network generated by IPA where CFL1 has a central role. (**A**) Protein-protein interaction network showing the differentially expressed proteins in IBS-D compared to healthy controls and Canonical Pathways (CP) and Biological Functions (Fx) associated to the proteins belonging to this particular network. The intensity of the node color indicates the degree of upregulation (red) or down regulation (green). Genes in uncolored nodes were not identified as differentially expressed in our proteomic study and were integrated into the computationally generated networks on the basis of the evidence stored in the IPA knowledge data base indicating relevance for this network. (**B**) Summary of the actin depolymerization pathway regulated by CFL1.

Sixteen healthy controls and 15 IBS-D patients were included in the study (Table 1). Proteomic profiling was performed first in a subset of subjects comprising 4 healthy controls and 4 IBS-D. Analysis of data revealed approximately 1,200 protein spots. By selecting abundance ratio of 1.3-fold as the threshold for the study, 139 spots were differentially produced between the two groups studied (P < 0.05). A search for protein identification was performed by introducing the obtained sequences from 139 spots in public databases, from which 64 proteins were identified (Fig. 1; Supplementary Table 2). IPA analysis revealed key pathways for intestinal homeostasis as playing an important role in IBS-D. Lipopolysaccharide (LPS)/interleukin 1 (IL1)-mediated alterations of nuclear retinoid x receptor (RXR) receptors, mitochondrial dysfunction and regulation of actin-based motility by Rho, appeared as the most significant pathways related to IBS-D proteomic profile (Table 2). Other significant pathways were also found to be related with alterations in actin-cytoskeleton function as clathrin-mediated endocytosis signaling, actin cytoskeleton signaling, caveolar-mediated endocytosis signaling and integrin signaling. In addition, the most significant protein network identified showed CFL1 as playing a key role by interacting with other proteins involved in the regulation of the actin cytoskeleton (Fig. 2A). As shown in Fig. 2B, CFL1 is tightly regulated by kinases and phosphatases activity. When CFL1 is phosphorylated in the serine 3 residue by TESK1 or LIMK, CFL1 is inactivated and, generally, translocated to the cell nucleus. As mentioned before, the activity of these kinases can be regulated by RhoGTPases (regulation of LIMK) or by integrin signaling through vinculin (VCL) (regulation of TESK1).

**Table 1:** Clinical and demographic characteristics of participants.

	**HC (n = 16)**	**IBS-D (n = 15)**	P-value
Gender (F:M)	8:8	9:6	—
Age (years; range)	26.1; (22–29)	39.3; (31–47)	0.002
Frequency of abdominal pain (mean; SD)	NA	5.7 ± 3.4	—
Severity of abdominal pain (median; CI)	NA	47.9; 95% CI [31.8–57.9]	—
Stool frequency (median; CI)	1.5; 95% CI [0.8–1.7]	3.0; 95% CI [2.5–4.9]	0.001
Bristol scale(mean; SD)	3.6 ± 0.7	5.1 ± 1.3	0.003

Note: IBS-D, diarrhea-predominant irritable bowel syndrome; HC, healthy controls; F, female; M, male; NA, non-applicable. SD: Standard deviation. CI: Confidence interval.

**Table 2:** Canonical signaling pathways most significantly associated with IBS-D protein expression profile.

**Ingenuity canonical pathways**	**Significance (P-value)**	Ratio*	**Molecules**
LPS/IL1 mediated Inhibition of RXR funtion	0.0003	2.94E-02	ALDH1B1, SULT1A3/SULT1A4, GSTT1, ALDH1A1, FABP1, SULT2A1
Mitochondrial Dysfunction	0.0003	3.94E-02	SDHA, PRDX3, SOD2, NDUFV2, PARK7
Regulation of Actin-based Motility by Rho	0.0007	4.55E-02	ACTR3, CFL1, ACTA2, ACTA1
VEGF Signaling	0.0008	4.26E-02	YWHAE, GRB2, ACTA2, ACTA1
Clathrin-mediated Endocytosis Signaling	0.0009	3.01E-02	ACTR3, TF, GRB2, ACTA2, ACTA1
RhoA Signaling	0.002	3.81E-02	ACTR3, CFL1, ACTA2, ACTA1
Bile acid biosynthesis	0.002	6.12E-02	ALDH1B1, AKR1A1, ALDH1A1
PI3K/AKT Signaling	0.002	3.03E-02	YWHAE, GRB2, YWHAB, YWHAZ
Actin Cytoskeleton Signaling	0.003	2.21E-02	ACTR3, CFL1, ACTA2, ACTA1
Caveolar-mediated Endocytosis Signaling	0.005	3.70E-02	ALB, ACTA2, ACTA1
Protein Ubiquitination Pathway	0.008	1.85E-02	PSMB3, PSME1, HSPA1A/HSPA1B, PSMB2, HSPA9
Fcγ Receptor-mediated Phagocytosis in Macrophages and Monocytes	0.01	3.19E-02	ACTR3, ACTA2, ACTA1
Integrin Signaling	0.01	1.99E-02	ACTR3, GRB2, ACTA2, ACTA1
Glucocorticoid Receptor Signaling	0.03	1.48E-02	HSPA1A/HSPA1B, GRB2, ANXA1, HSPA9
Ephrin Receptor Signaling	0.05	1.55E-02	ACTR3, CFL1, GRB2

^*^Ratio: number of molecules in the analysis that are associated with the canonical pathway divided by the total number of genes that map to the canonical pathway.

### Integrated protein/gene expression analysis reveals convergence of canonical pathways, biological functions and disease mechanisms in the jejunal mucosa of IBS-D

**Figure 3:**
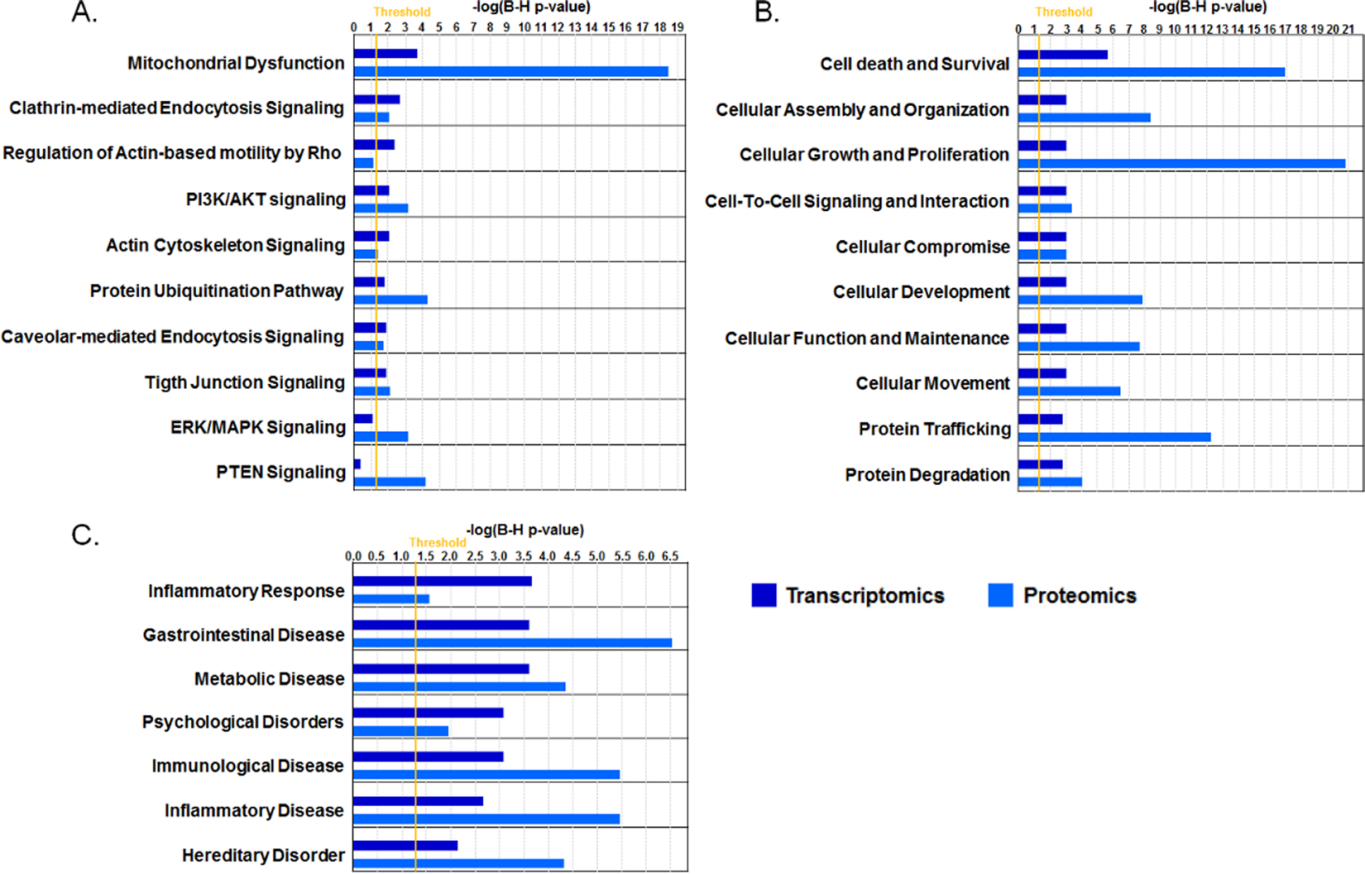
IPA comparative analysis of microarray and proteomic expression profiles in IBS-D. (**A**) Canonical signaling pathways; (**B**) Biological functions; and (**C**) Diseases and disease mechanisms most significantly associated with IBS-D transcriptomic (dark blue) and proteomic (light blue) profiles. The significance value associated with each category is a measure of the likelihood that the association between dysregulated transcripts and a given category is due to random chance. The x-axis of each graph shows the significance, expressed as the negative exponent of the P-value calculation for each category, increasing with bar height. The orange line shows a threshold P-value of 0.05.

In a previous work, we observed differential mucosal gene expression when comparing healthy controls with IBS-D by microarray technology^6^. In the present study, there was a discrete overlap between differentially expressed genes and proteins in IBS-D patients; however, using an integrated analysis of both expression profiles, we identified common signature pathways, biological functions and disease mechanisms associated with IBS-D (Fig. 3). In particular, cellular assembly and cell-to-cell signaling were the most significant biological functions associated with IBS-D (Fig. 3B). Moreover, immunological, gastrointestinal and inflammatory diseases were the most relevant disease mechanism associated with both IBS-D transcriptional profiles (Fig. 3C).

### Decreased TESK-1 mediated CFL1-phosphorylation in IBS-D patients

**Figure 4:**
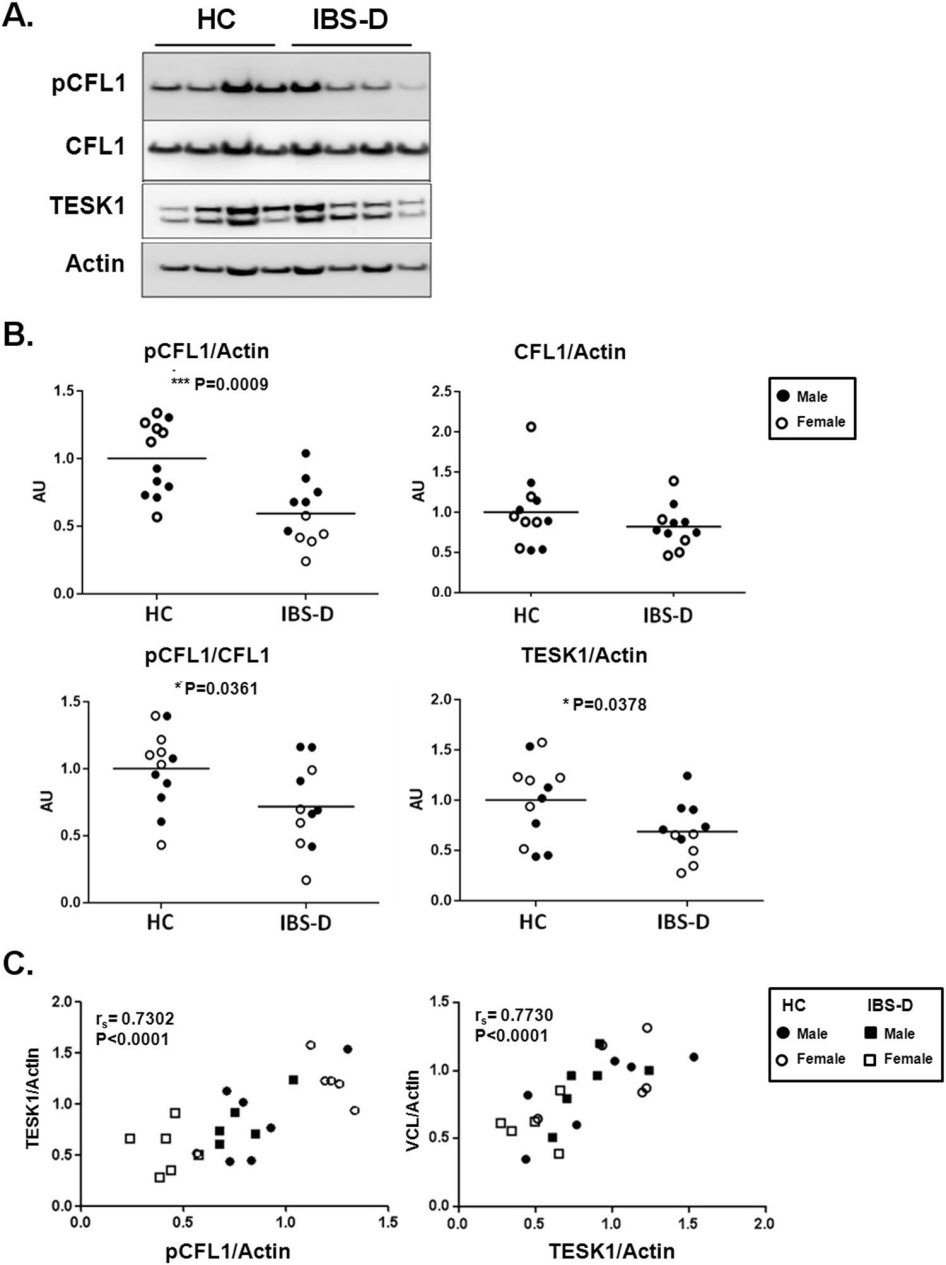
Expression analysis CFL1 signaling pathway in IBS-D. (**A**) Representative Western blot image showing expression of pCFL1, CFL1, TESK1 in the jejunal mucosa of four controls and four patients with IBS-D. (**B**) Normalization was performed using actin as loading control. Bands were quantified and results are expressed as fold-change respect to the average of the control group. Comparisons were performed by the Mann-Whitney U test (P-values shown). (**C**) Correlation between the CLF1 phosphorylation levels and VCL expression with TESK1 expression performed by Spearman correlation (r_s_ and P-values shown). HC are represented with circles and IBS-D patients with squares. AU: arbitrary units.

Regulation of actin-based motility by Rho is significantly altered at both transcriptomic and proteomic levels in IBS-D (Fig. 3A) which is consistent with our previous findings showing cytoskeleton alterations in IBS-D patients^7^. In addition, 28% of the identified altered proteins in IBS-D patients are involved in the dynamic regulation of actin depolymerization events (Fig. 1, Fig. 2A and Supplementary Table 2) where CFL1 plays a major role (Fig. 2). In order to validate our findings, we have further explored the molecular mechanisms by which actin rearrangement and cytoskeleton disruption are linked to the loss of intestinal barrier integrity observed in IBS-D patients^7^ by analyzing expression of actin-binding proteins in a larger cohort of patients and controls. CFL1-mediated actin polymerization regulation is tightly modulated by phosphorylation events driven by specific kinases and phosphatases, (summarized in Fig. 2B). No differences in protein expression of total CFL1 were observed (Supplementary Figure 2, Fig. 4A,B); however, phosphorylated CFL1 (pCFL1) was reduced in IBS-D patients (Supplementary Figure 2, Fig. 4A,B). In concordance, the CFL1 specific kinase TESK1 was down-regulated in IBS-D patients (Supplementary Figure 2, Fig. 4A,B) and this down-regulation positively correlated with pCFL1 expression level (Fig. 4C). No significant differences in other CFL1 regulatory proteins were found (Supplementary Figure 3). In addition, TESK1 can regulate the localization and expression of VCL, which is the protein connecting actin filaments with integrins^18^. Expression levels of TESK1 correlate with VCL, however, we did not observe significant differences in VCL comparing healthy controls with IBS-D (Fig. 4C).

### Differential regulation of CFL1-actin activation by sex and disease

**Figure 5:**
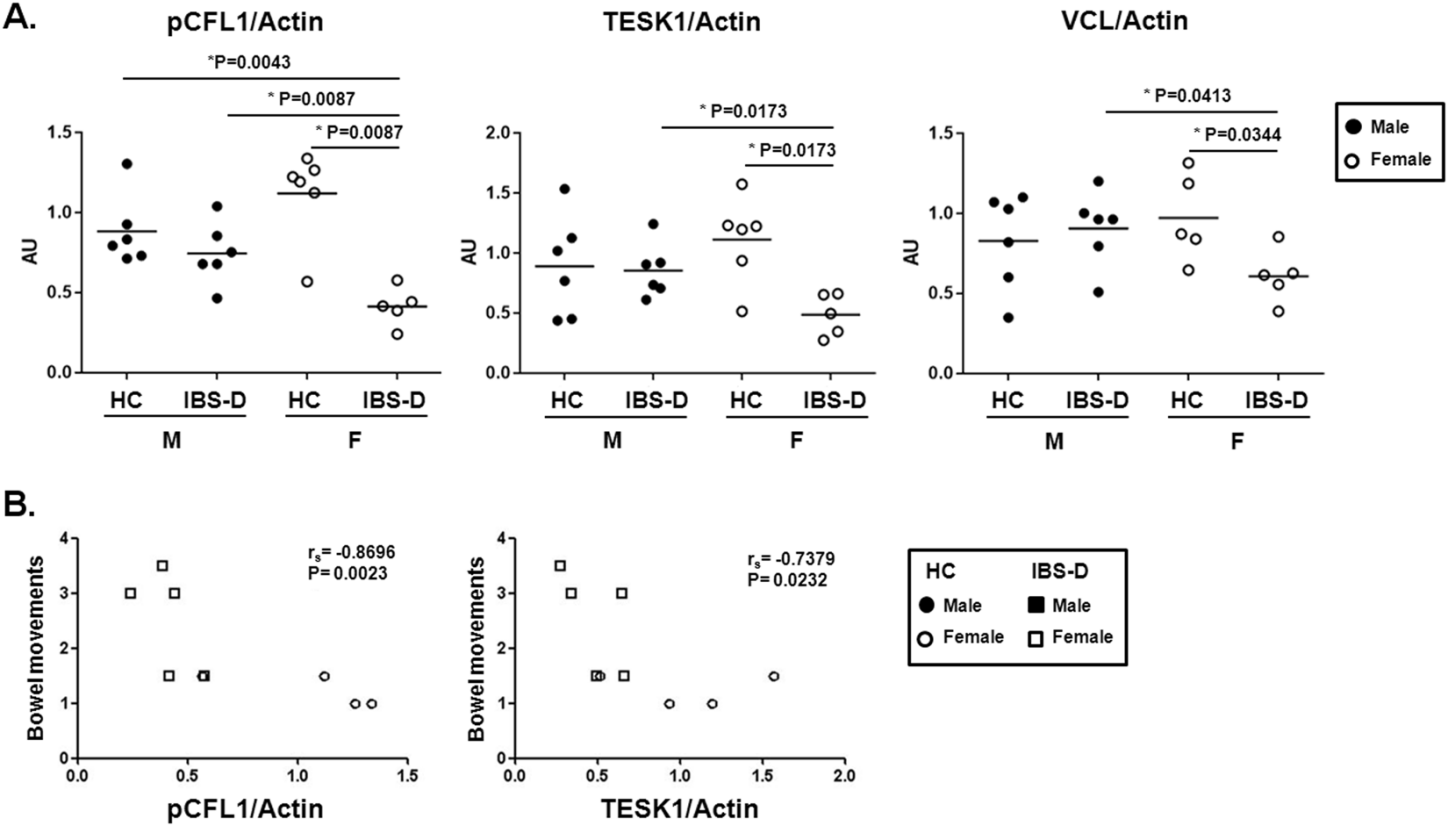
Expression analysis CFL1 signaling pathway in IBS-D samples divided by sex. (**A**) Normalization was performed using actin as loading control. Bands were quantified and results are expressed as fold-change respect to the average of the control group. Comparisons were performed by the Mann-Whitney U test (P-values shown). (**B**) Correlation between pCFL1 and TESK1 levels and number of bowel movements performed by a Spearman correlation (r_s_ and P-values shown). HC females are represented with circles and IBS-D female patients with squares. AU: arbitrary units.

To understand the clinical relevance of the molecular changes observed in the CFL1 pathway, we first analyzed the global association between pCFL1 and TESK1 expression with IBS-D major symptoms; however, no significant correlations were found (Supplementary Table 3). Interestingly, when stratifying groups by sex, we observed that female patients showed lower expression levels of pCFL1 and TESK1 compared to male IBS-D patients and healthy controls (Fig. 5A). Furthermore, the expression of these proteins negatively correlated with bowel movements in female subjects (Supplementary Table 3 and Fig. 5B).

## Discussion

In the present study, we demonstrate that the jejunum of IBS-D patients has a distinctive proteomic profile compared to healthy controls. As an extension of our previous research, the integrative analysis of gene and protein expression profiles performed here further supports prior studies describing the loss of intestinal epithelial barrier function as a central molecular mechanism in the disease^6,7,10,21,23,27^. In addition, we reveal for the first time the involvement of TESK1 in human disease, making this protein a potential novel candidate for diagnosis and treatment strategies in IBS.

Proteomic approaches have been previously applied to study the colonic mucosa of experimental models mimicking IBS  clinical manifestations^25,26^ and, more recently, Goo *et al*. reported a distinctive proteomic profile in urine samples from IBS patients^24^. Having studied biological samples from such a different origin (urine *vs*. tissue, rodent *vs*. human) it is reasonable to expect no common individual proteins across studies. However, common pathways and functions (regulation of actin-based motility and actin cytoskeleton signaling) are identified in both, urine and jejunal tissue, proving the consistency of our data and suggesting that these pathways may represent novel candidates for biomarker discovery in IBS. However, further studies comparing tissue and urine samples from the same subject will be needed in order to validate proteins from these pathways as potential biomarkers.

The actin cytoskeleton is a highly dynamic structure that undergoes rearrangement (polymerization and depolymerization) under the control of various actin-binding proteins^28,29^, including members of the CFL1 family and other actin-depolymerizing proteins^30,31^. Although a decreased expression of total CFL1 in IBS-D could not be validated in our study, we observed lower levels of the inactive form of CFL1 (the phosphorylated protein, pCFL1)^32,33^. Reduced pCFL1 would result in increased actin depolymerization events compromising, therefore, intestinal epithelial integrity through tight junctions dissassembly and internalization of occludin^34,35^, alterations previously described in the jejunum of IBS-D patients^7^. CFL1 phosphorylation is driven by four specific kinases LIMK 1 and 2, and TESK1 and 2^16,17^, and counter-regulated by phosphatases, as slingshot and chronophin/PDXP^36,37^. Although LIMKs are the most studied kinases in CFL1 inactivation, these proteins were not altered in IBS-D samples. On the contrary, TESK1 was down-regulated in patient samples and correlated with reduced pCFL1, suggesting that TESK1 is a major kinase driving CFL1 inactivation in the small bowel mucosa. Another important pathway regulating CFL1 phosphorylation is ROCK-dependent LIMK activation^38^. Although we did not observe differences in total expression of ROCK and LIMK, the activity of this pathway could still be depressed due to inactivation of Rho by upstream mechanisms that have not been investigated in the present study. In addition, a crucial binding partner of CFL1, WDR1 (also known as actin-interacting protein-1), was also identified by our proteomic approach. WDR1 is essential for actin depolymerization kinetics through interaction with CFL1^39^. Indeed, a recent study directly implicated WDR1 in the regulation of intercellular junctions and barrier properties of intestinal epithelial cell monolayers^40^. Hence, upregulation of WDR1 could interact with CFL1 dephosphorylation to stimulate F-actin turnover and destabilize junction-associated actin cytoskeleton in the intestinal mucosa in IBS-D. Alterations in CFL1 pathway may, therefore, be involved in the complex regulatory network of molecular mechanisms underlying increased intestinal epithelial permeability described in IBS-D. Remarkably, although TESK1 has been described to play a key role in integrin-mediated actin reorganization through phosphorylating and inactivating CFL1 and it has been found expressed in epithelial colon cancer cells^41^, no studies so far have provided evidence of a major role of this protein in any human disease mechanism. Our study represents the first report showing the association of TESK1 expression in human disease, in particular in female subjects. Indeed, a remarkable finding in this work is the identification of sex-dependent differences in CFL1 regulation. In particular, phosphorylation of CFL1 and expression levels of TESK1 are lower and correlated with bowel habits in female IBS-D patients. However, these results should be taken with caution, since the number of subjects is relatively low in each group. Although the relation between sex and cytoskeleton alterations is not clear, a possible explanation could be found in the role of female sexual hormones in the modulation of actin dynamics^42^. Indeed, estradiol regulates actin cytoskeletal dynamics in the hippocampus and endothelium through ROCK and Rac/p21-activated kinase signaling pathways^43,44^. In addition, estrogen treatment increases pCFL1 and, in consequence, actin polymerization events^45^. The connection between actin cytoskeleton rearrangements and sex could, therefore, help to shed some light on the mechanisms associated to the female bias that exist in IBS and, indeed, merit further investigation.

In addition to the actin cytoskeleton-related pathways, LPS/IL1 mediated alterations of nuclear RXR receptors and mitochondrial dysfunctions were identified by IPA analysis with higher significance. Higher concentration of both LPS and IL1 have been previously reported in IBS^46–49^ and related to alterations in permeability in cell culture models^46^. IBS patients have also showed higher expression of IL1 alpha and beta in the duodenum, which correlate with small intestinal bacterial overgrowth, bloating, and diarrhea. Furthermore, IL1beta concentration was higher in peripheral blood mononuclear cells supernatants from IBS-D, compared to healthy controls and IBS with constipation, and related with direct stimulation of colonic afferents via TTX-sensitive ion channels and mechanical hypersensitivity. On the other hand, mitochondrial dysfunction is an interesting finding in the jejunal mucosa of IBS-D patients. In this line, we have described significant alterations in mitochondrial enzymatic expression, activity, and morphology in the IBS-like model of crowding stress^50,51^. These findings may be linked to the cytoskeleton alterations we have previously described in IBS-D^7^ due to the ATP dependence of the actin polymerization homeostasis which make this process vulnerable to alterations in mitochondrial function^30^. In fact, estrogen stimulation of epithelial breast cancer cells lead to mitochondrial reactive oxygen species production, which can be increased with the actin disruption and integrin activation^52^. These effects link our observations, but more studies will be necessary to connect all these pieces together.

Finally, we would like to acknowledge the limitations associated with the approach taken in this study. First, the correlation between transcriptome and proteome data is low. Indeed, the transcriptomic and proteomic independent analysis do not match in the same genes/proteins, however, by integrating data, we identify the same biological pathways and functions. In fact, this multi-omic strategy points to a robust effect of the CFL1 pathway regulating cytoskeleton dynamics and epithelial permeability in IBS-D^14,53,54^. In addition, it should be taken into account that none of the high-throughput techniques applied here is able to reflect the post-translational events like protein degradation rates and phosphorylation and cellular localization which are known to alter protein half-lives and their detection. Indeed, all three post-translational mechanisms (proteasome degradation, phosphorylation and protein delocalization) have been identified to affect proteins involved in intestinal epithelial integrity in IBS, nor in this study neither in previous reports^6,7,23,55^. In addition, and related to the former point, several critical proteins involved in intestinal epithelial integrity are absent from our proteomic data. In particular, proteins within the major networks responsible for apical junction stabilization and actin dynamics. This may reflect technical issues with the sensitivity of the proteomics methods available and parameters (for example, false negative and low maximum mass range for peptides with post-translational modifications). Indeed, we took a non-targeted proteomics approach which has certain technical limitations. However, using a more targeted approach, as we did previously for the apical junctional proteins core proteins^7^ and for TESK1 and other proteins belonging to the CFL1 pathway here, we could identify alterations in these specific proteins in the jejunal mucosa.

In conclusion, our findings support an altered regulation of cytoskeleton dynamics in the small bowel mucosa of IBS-D, particularly in female patients. We show evidence of the involvement of a novel mechanism in bowel dysfunction by TESK1-mediated regulation of CFL1 pathway which may contribute to the female predisposition to suffer IBS. Further understanding of the mechanisms involving sex-mediated regulation of mucosal epithelial integrity may have significant preventive, diagnostic, and therapeutic implications for IBS.

## Material and Methods

### Participants

Recently diagnosed, naïve IBS-D patients meeting the Rome III criteria^56^ were prospectively recruited from the outpatient gastroenterology clinic. Healthy control subjects were recruited by public advertising, as a control group. Prior to inclusion, a complete medical history and physical examination was carried out in all participants. Food allergy was ruled out using a battery of skin prick tests (Leti, Madrid, Spain) for 32 common food allergens with histamine and saline as positive and negative controls, respectively. Candidates with either positive reaction by skin prick tests or clinical history consistent with food allergy (digestive and/or extra-digestive symptoms associated with exposure to certain food components) were excluded. Other exclusion criteria included age <18 or >70 years, major psychiatric and organic diseases, past episodes of infectious gastroenteritis and gastrointestinal comorbidities, drug or pharmaceutical compound intake in the last 2 weeks, steroids, anti-allergic or immunosuppressive and related drugs in the last 3 months and radiotherapy or chemotherapy in the last 6 months. Written informed consent was obtained from each participant and data was stored in an anonymous fashion according to the approval from the ethics committee of the Institut de Recerca Hospital Universitari Vall d´Hebron (PR(AG)221/2015). All methods were performed in accordance with our institution guidelines and regulations.

### Clinical assessment

The following parameters were recorded in all participants using daily questionnaires over a 10-day period before the biopsy: a) the severity of abdominal pain by a 100-point visual analogue scale; b) the frequency of abdominal pain (number of days with pain); c) the stool frequency (number of bowel movements/day); d) the stool consistency by the Bristol stool form score^57^.

### Experimental design and procedures

In order to narrow down the molecular pathways involved in the origin of IBS-D patients we have combined part of our previously published transcriptomic data^7^ (data set available at the NCBI Gene Expression Omnibus http://www.ncbi.nlm.nih.gov/geo; accession number GSE14841) with the proteomic profile we have generated in this study. Common pathways and biological functions were subsequently identified using IPA and the most relevant candidates were then further followed-up using complementary and more sensitive approaches in a higher number of patients and controls. Ultimately, obtained data was correlated with clinical data from patients and controls in order to understand the significance of our results.

Tissue samples were obtained as follows. A single mucosal biopsy per participant was obtained from the proximal jejunum, 5–10 cm distal to the Treitz’s angle, using a modified Watson’s capsule as described previously^58^. Tissue samples were immediately snap frozen in liquid nitrogen for protein analysis as described below.

The following analyses were subsequently performed (Supplementary Figure 1). Detailed description of procedures is given in the supplementary material and methods section.

- Proteomic profiling by two-dimension difference in-gel electrophoresis (2D-DIGE) analysis: total protein was isolated from jejunal biopsies of a discovery cohort comprising four healthy controls and four IBS-D. Protein lysates were labeled with either Cy3 or Cy5 cyanine dye to distinguish proteins from healthy controls or IBS-D and 2D-DIGE was performed with pooled samples. Cy3 and Cy5 images were scanned and analyzed for determination of significant alterations in protein abundances using the DeCyder V. 5.0 software^59^.
- Protein identification and data analysis: protein spots with higher differences in expression were picked from the gel and digested with trypsin^60^. Peptide mass fingerprint of proteins was obtained by MALDI-TOF-TOF mass spectrometry and identification through database searches were performed using by Mascot algorithm^61^. Identified proteins were submitted to the IPA software for the pathway analysis^6^.
- Validation of profiling results by western blot: for validation, we included 12 additional controls and 11 additional IBS-D patients. Protein homogenates were separated by electrophoresis and blotted to a polyvinylidene fluoride (PVDF) membrane. Detection of proteins belonging to specific pathways was performed with primary antibodies listed in Supplementary Table 1. All blots were probed with anti-β-actin antibody as a protein loading control. The bands were scanned and quantitated by densitometry (ImageJ 1.50 software) on the same immunoblot. Values were normalized to β-actin expression and fold-change was calculated for each sample respect to the average of the control group.

### Pathway and network analysis

To identify relevant biological pathways implicating those genes and proteins differentially expressed, we applied the IPA methodology (IPA Software, Ingenuity® Systems, www.ingenuity.com). IPA integrates selected omics data sets (genomics, transcriptomics, proteomics) with mining techniques to predict functional connections and their interpretation in the context of protein networks that comprise protein-protein interactions and related biological functions and canonical signaling pathways. Two types of analysis were performed in our omics data. Further details are given in the supplementary material and methods.

1. Proteomic core analysis to identify the most relevant biological functions and pathways associated to the differentially expressed proteins followed by protein network analysis. For network analysis, IPA computed a score (P-score = −log10(P-value)) according to the fit of the set of supplied genes and a list of biological functions stored in the IPKB. The score takes into account the number of proteins in the network and the size of the network to approximate how relevant this network is to the original list of genes and allows the networks to be prioritized for further studies. In addition, the functions and pathways associated with the proteins in the networks are also identified.
2. Transcriptomic (from reference^6^) and proteomic integrative analysis to identify common biological functions and pathways.

### Statistical analysis

Statistical analysis of proteomic data: image analysis and statistical quantification of relative protein abundances were performed using DeCyder V. 6.0 software (GE Healthcare). Differentially expressed proteins were selected applying a 1.3 fold threshold and P < 0.05.

Statistical analysis for western blot: two-tailed parametric or non-parametric tests were used as appropriate (unpaired Student’s t-test, Mann-Whitney U test, respectively) using GraphPad Prism 5.0 software. Relationship between clinical variables and gene expression were assessed by Spearman’s correlation rho. Data are expressed as mean ± standard deviation (SD), unless otherwise stated.

### Grant Support

Supported in part by Fondo Europeo de Desarrollo Regional (FEDER), Fondo de Investigación Sanitaria and Centro de Investigación Biomédica en Red de Enfermedades Hepáticas y Digestivas-CIBERehd, Instituto de Salud Carlos III, Subdirección General de Investigación Sanitaria, Ministerio de Economía y Competitividad: CD15/00010 (B. Rodiño-Janeiro), IJCI-2015-26099 (C. Martínez), FI12/00254 (E. Salvo-Romero), PI12/00314 & PI15/00301 (C. Alonso-Cotoner), PI11/00716 & PI14/00994 (J. Santos), CPII16/00031, PI13/00935 & PI16/00583 (M. Vicario); CIBER-EHD CB06/04/0021 (FA, CAC, JS, MV); Ministerio de Educación, Dirección General de Investigación: SAF 2016-76648-R (F. Azpiroz); Agència de Gestió d’Ajuts Universitaris i de Recerca, de la Generalitat de Catalunya: 2014 SGR 1285 (F. Azpiroz); Vall d’Hebron Institut de Recerca, Programa de becas predoctorales “Amics de Vall d’Hebron”: PRED-VHIR-2014-018 (M. Fortea), PRED-VHIR-2016-34 (C. Pardo-Camacho).

## Electronic supplementary material

Suplementary information
