# Supplementary material for: Decreased TESK1-mediated cofilin 1 phosphorylation in the jejunum of IBS-D patients may explain increased female predisposition to epithelial dysfunction
Source: Sci Rep. 2018 Feb 2;8:2255. doi: 10.1038/s41598-018-20540-9 (PMC5797119; doi:10.1038/s41598-018-20540-9)
Supplement: Supplementary file 1 — Suplementary information [file 41598_2018_20540_MOESM1_ESM.pdf]

Decreased TESK1-mediated cofilin 1 phosphorylation in the jejunum of IBS-D patients may explain increased female predisposition to epithelial dysfunction.

Bruno K. Rodiño-Janeiro[1*†], Cristina Martínez[1†], Marina Fortea[2], Beatriz Lobo[1], Marc Pigrau[1], Adoración Nieto[1], Ana María González-Castro[2], Eloísa Salvo-Romero[1,2], Danila Guagnozzi[2], Cristina Pardo-Camacho[2], Cristina Iribarren[2], Fernando Azpiroz[1,3], Carmen Alonso-Cotoner[1,3†], Javier Santos[1,3*†], Maria Vicario[2,3†].

[1]Laboratories of Neuro-Immuno-Gastroenterology and [2]Translational Mucosal Immunology, Digestive System Research Unit, Vall d'Hebron Institut de Recerca; Department of Gastroenterology, Hospital Universitari Vall d'Hebron, Universitat Autònoma de Barcelona (Facultat de Medicina), Barcelona, Spain; [3]Centro de Investigación Biomédica en Red de Enfermedades Hepáticas y Digestivas (CIBERehd). [†]Equal contribution

***Corresponding authors**: Bruno Kotska Rodiño-Janeiro, PhD, Laboratory of Neuro-immuno-gastroenterology, Digestive System Research Unit, Vall d'Hebron Institut de Recerca, Department of Gastroenterology, Hospital Universitario Vall d'Hebron. Paseo Vall d'Hebron 119-129, Barcelona, Spain. Tel. +34 93 489 4035 E-mail: bruno.rodino@vhir.org

Javier Santos Vicente, MD, PhD, Laboratory of Neuro-immuno-gastroenterology, Digestive System Research Unit, Vall d'Hebron Institut de Recerca, Department of Gastroenterology, Hospital Universitario Vall d'Hebron. Paseo Vall d'Hebron 119-129, Barcelona, Spain. Tel. +34 93 489 4035 E-mail: javier.santos@vhir.org

Supplementary material and methods

<u>Protein isolation and DIGE analysis</u>

Samples were homogenized in the FastPrep homogenizer (MP Biomedicals, Ohio, USA) in T-PER buffer and protease and phosphatase inhibitor cocktail (ThermoFisher Scientific, Massachusetts, USA), following manufacturer recommendations. The supernatant was sonicated four times for 10 seconds at 4°C and insoluble material was removed by centrifugation (350 g, 5 min, 4°C). Isolated protein samples were then subjected to a modified

acetone-trichloroacetic acid precipitation (2D-CleanUp kit; GE Healthcare, Maryland, USA). The protein pellets were resuspended in the DIGE labeling buffer (8 M urea, 4% [wt/vol] 3-[(3-cholamidopropyl)-dimethylammonio]-1-propanesulfonate [CHAPS], 30 mM Tris [pH 8.0]), and the protein concentration was determined using the Bio-Rad RCDC protein assay as described by the manufacturer (Bio-Rad, California, USA). The protein concentration in the samples was then adjusted to 2 mg/mL by addition of DIGE labeling buffer. Fifty micrograms of each sample was labeled with either Cy3 or Cy5 cyanine dye (GE Healthcare), following general procedures.

Two-dimension electrophoresis was performed using GE Healthcare reagents and equipment. First-dimension isoelectric focusing was performed on immobilized pH gradient strips (24 cm; pH 3 to 10 or 4 to 7) using an Ettan IPGphor system. Samples were applied via cup loading near the basic ends of the strips, which were previously rehydrated overnight in 450 µL of rehydration buffer (8 M urea, 4% [wt/vol] CHAPS, 1% Pharmalytes [pH 3 to 10], 100 mM DeStreak). After focusing for a total of 67 kV-h, the strips were equilibrated first for 15 min in 6 ml of reducing solution (6 M urea, 100 mM Tris-HCl [pH 8], 30% [vol/vol] glycerol, 2% [wt/vol] SDS, 5 mg/mL DTT) and then for a further 15 min in 6 mL of alkylating solution (6 M urea, 100mM Tris-HCl [pH 8], 30% [vol/vol] glycerol, 2% [wt/vol] SDS, 22.5 mg/mL iodoacetamide) on a rocking platform. Second-dimension SDS-polyacrylamide gel electrophoresis was run by overlaying the strips on 12.5% isocratic Laemmli gels (24 by 20 cm), cast in low-fluorescence glass plates, on an Ettan DALT VI system. Gels were run at 20°C and at a constant power of 2.5 W per gel for 30 min, followed by 17 W per gel until the bromophenol blue tracking front had run off the bottoms of the gels (about 5 h).

Fluorescence images of the gels were acquired on a Typhoon 9400 scanner (GE Healthcare). Cy3 and Cy5 images were scanned at 532 nm excitation/580 nm emission and 633 nm excitation/670 nm emission, respectively, at a 100 µm resolution. Image analysis and determination of significant alterations in protein abundances were performed automatically with the DeCyder V. 5.0 software (GE Healthcare).

<u>Protein identification and data analysis</u>

Protein spots of interest were excised from the gel by using an automated Spot Picker (GE Healthcare). In-gel trypsin digestion was performed essentially as previously described[1], using

autolysis-stabilized trypsin (Promega, Wisconsin, USA). Tryptic digests were purified using ZipTip microtiter plates (Millipore, Carrigtwohill, Ireland).

Matrix-assisted laser desorption ionization mass spectrometric analysis of tryptic peptides was performed on an Ultraflex time-of-flight/time-of-flight (TOF-TOF) instrument (Bruker, Bremen, Germany). Samples were prepared using α-cyano-4-hydroxycinnamic acid as a matrix on anchor chip targets (Bruker). Identification of the proteins was carried out by peptide mass fingerprint data and/or by TOF-TOF PSD. Database searches were performed using the Mascot algorithm (Matrix Science, London, UK). Identified proteins where then submitted to Ingenuity Pathway Analysis (IPA) Software 7.0 as previously described[2].

Western blot

Jejunal biopsies were homogenized using FastPrep (MP Biomedicals) in TRIzol. Protein extractions from the organic phase of TRIzol were performed following the manufacturer instructions (ThermoFisher Scientific). Protein quantifications were performed using Quick Start™ Bradford Protein Assay (Biorad). Equal amounts of protein were separated by NuPAGE® Novex® 4-12% Bis-Tris Protein Gels (ThermoFisher Scientific). Primary antibodies and dilutions used are listed in supplementary table 1. Peroxidase-conjugated secondary antibody and the chemiluminescence detection system SuperSignal West Femto (ThermoFisher Scientific) were used to detect bound antibodies. All blots were probed with mouse antihuman β-actin as a protein loading control. Image acquisition was performed with a LAS-3000 Imaging System From Fuji. Densitometric comparison was carried out on the same immunoblot using the ImageJ software (National Institutes of Health; http://rsb.info.nih.gov/ij/).

Proteomic core analysis

The list of proteins obtained in profiling experiments was overlaid onto a global molecular network developed from information contained in the IPA knowledge base (IPKB). For network analysis, IPA computed a score (P-score=-log10(P-value)) according to the fit of the set of supplied genes and a list of biological functions stored in the IPKB. The score takes into account the number of proteins in the network and the size of the network to approximate how relevant this network is to the original list of genes and allows the networks to be prioritized for further studies. A score >3 (P<0.001) indicates a >99.9% confidence that a network was not generated by chance alone. The network identified is presented as a graph indicating the molecular

relationships between proteins. Moreover, networks are preferentially enriched for proteins with the most extensive interactions, and for which interactions are specific with the other proteins in the network (rather than proteins that are promiscuous, those that interact with a broad selection of proteins throughout IPKB).

The functional analysis identified the biological functions and the canonical signaling pathways that were most significant to the input data set. The significance of the association between the input data set and the functions or pathways was determined based on two parameters: (1) a ratio of the number of proteins from the data set that map to the function/pathway divided by the total number of proteins that map to the function/pathway and (2) a P value calculated using Fischer's exact test determining the probability that the association between the proteins in the dataset and the function/pathway is explained by chance alone.

Protein/mRNA pairing analysis

This combined analysis of protein and mRNA profiles was carried out by investigating the coexpression profile of protein-mRNA pairs.

References

1 Shevchenko A, Wilm M, Vorm O, *et al.* Mass spectrometric sequencing of proteins silver-stained polyacrylamide gels. *Anal Chem* 1996;**68**:850–8.

2 Martínez C, Vicario M, Ramos L, *et al.* The jejunum of diarrhea-predominant irritable bowel syndrome shows molecular alterations in the tight junction signaling pathway that are associated with mucosal pathobiology and clinical manifestations. *Am J Gastroenterol* 2012;**107**:736–46. doi:10.1038/ajg.2011.472

3 Martínez C, Lobo B, Pigrau M, *et al.* Diarrhoea-predominant irritable bowel syndrome: an organic disorder with structural abnormalities in the jejunal epithelial barrier. *Gut* 2013;**62**:1160–8. doi:10.1136/gutjnl-2012-302093

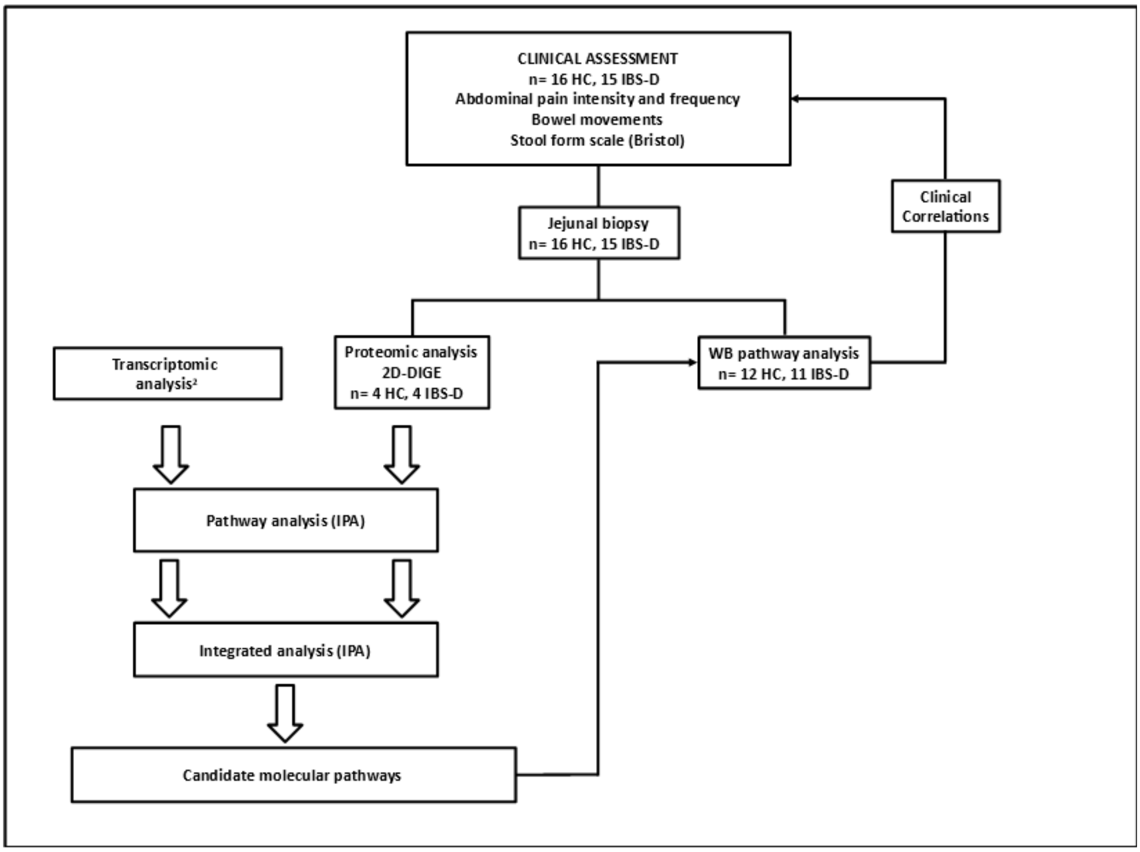

Figure supplementary 1. Experimental design: workflow of the human samples analysis.

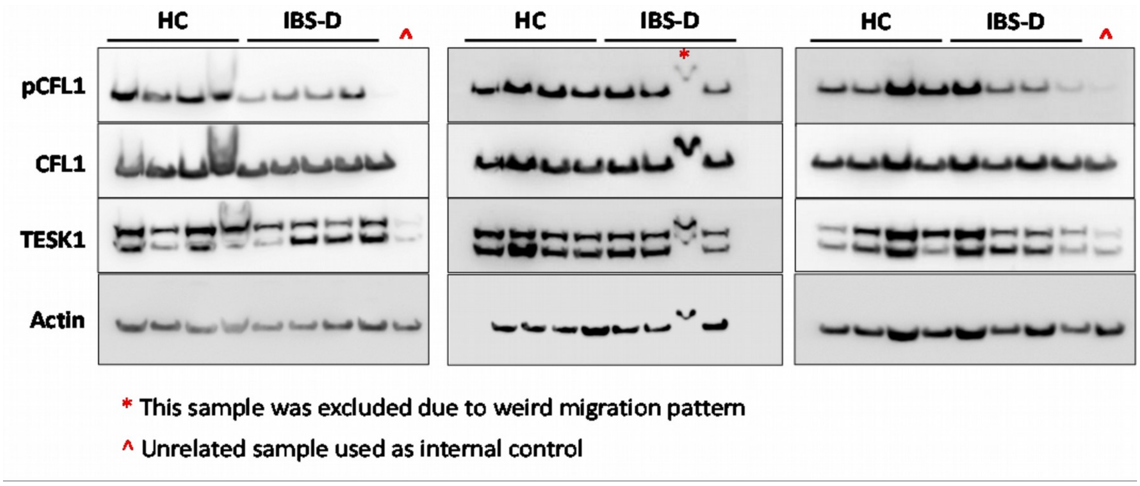

Figure supplementary 2:A. Western blot images showing expression of pCFL1, CFL1, TESK1 in the jejunal mucosa of 12 HC and 11 patients with IBS-D. B. Normalization was performed using actin as loading control. Bands were quantified and results are expressed as fold-change respect to the average of the HC group.

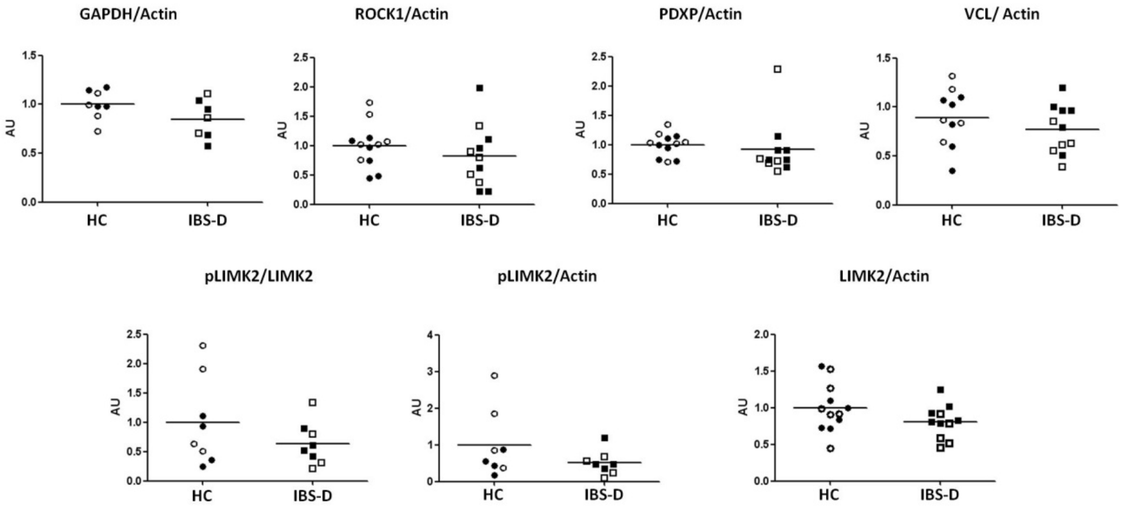

Figure supplementary 3. Protein fold-change of GAPDH, ROCK1, PDXP, VCL, pLIMK2, LIMK2 calculated for each sample with reference to the average of the target protein to actin or LIMK2 ratio of the healthy control group. Comparisons were performed by the Mann-Whitney U test.

Supplementary table 1. List of antibodies used for the western blot protein identification. mAb: monoclonal antibody, pAb: polyclonal antibody.

**Antibody**	**Manufacturer /Distributor**	**Dilution**	**Secondary (1:5,000)**	**Secondary Manufacturer/Distributor**
Cofilin (D3F9) XP® Rabbit mAb	Cell signaling technologies (5175S)	1:1,000	Anti-rabbit IgG, HRP-linked Antibody	Cell signaling technologies (7074P)
Phospho-Cofilin (Ser3) (77G2) Rabbit mAb	Cell signaling technologies (3313S)	1:1,000	Anti-rabbit IgG, HRP-linked Antibody	Cell signaling technologies (7074P)
Phospho-LIMK1 (Thr508)/LIMK2 (Thr505) Rabbit pAb	Cell signaling technologies (3841S)	1:1,000	Anti-rabbit IgG, HRP-linked Antibody	Cell signaling technologies (7074P)
LIMK2 (8C11) Rabbit mAb	Cell signaling technologies (3845S)	1:1,000	Anti-rabbit IgG, HRP-linked Antibody	Cell signaling technologies (7074P)
TESK1 (D49D4) Rabbit mAb	Cell signaling technologies (4655S)	1:1,000	Anti-rabbit IgG, HRP-linked Antibody	Cell signaling technologies (7074P)
ROCK1 (C8F7) Rabbit mAb	Cell signaling technologies (4035S)	1:1,000	Anti-rabbit IgG, HRP-linked Antibody	Cell signaling technologies (7074P)
Chronophin/PDXP (C85E3) Rabbit mAb	Cell signaling technologies (4686S)	1:1,000	Anti-rabbit IgG, HRP-linked Antibody	Cell signaling technologies (7074P)
β-Actin Mouse mAb	Sigma Aldrich (A5441)	1:10,000	Goat anti-Mouse IgG (H+L) HRP conjugate	ThemoFisher Scientific (32430)
Vinculin Mouse mAb	Sigma Aldrich (V9264)	1:30,000	Goat anti-Mouse IgG (H+L) HRP conjugate	ThemoFisher Scientific (32430)
GAPDH mouse mAb	Santa cruz Biotechnology (sc-365062)	1:1,000	Goat anti-Mouse IgG (H+L) HRP conjugate	ThemoFisher Scientific (32430)

Supplementary table 2. Differentially expressed spots identified in IBS-D patients compared with the healthy volunteer group. Uniprot accession number of proteins and their fold change expression are indicated. In yellow, proteins related with actin dynamics and polymerization. Proteins with a fold change higher than 1,5 or -1,5 are represented in green or red, respectively.

P-value	Foldchange	Identification	# access (Uniprot)	Gene name
2,61E-04	1,91	Isoform 1 of Adseverin	Q9Y6U3	SCIN
0,004	1,69	Aconitate hydratase, mitochondrial	Q99798	ACO2
7,48E-04	1,69	Actin-like protein 3	P61158	ACTR3
0,004	1,69	ALB protein (Serum albumin)	P02768	ALB
0,005	1,63	Gamma-tubulin complex component 2 (83 Kda Protein)	Q9BSJ2	TUBGCP2
0,001	1,59	WD repeat-containing protein 1	O75083	WDR1
1,38E-04	1,58	Keratin, type I cytoskeletal 18	P05783	KRT18
0,014	1,55	Serum albumin	P02769	ALB
0,001	1,53	KRT8 protein	Q969I0	KRT8
0,008	1,49	Serotransferrin precursor	P02787	TF
0,001	1,43	Protein transport protein Sec23A	Q15436	SEC23A
0,004	1,42	Guanine deaminase	Q9Y2T3	GDA
3,59E-04	1,42	Heat shock 70 kDa protein 1	P08107	HSPA1A
0,004	1,38	GDP-mannose 4,6 dehydratase	O60547	GMDS
0,002	1,34	Elongation factor 2	P13639	EEF2
0,025	1,34	Selenium-binding protein 1	Q13228	SELENBP1
0,003	1,31	Retinal dehydrogenase	P00352	ALDH1A1
0,002	1,3	Stress-70 protein, mitochondrial	P38646	HSPA9
0,011	1,3	Keratin, type II cytoskeletal 8	P05787	KRT8
0,003	1,28	Lamin-B1	P20700	LMNB1
0,002	1,27	Eukaryotic initiation factor 4A-II	Q14240	EIF4A2
0,003	1,25	Keratin, type I cytoskeletal 20	P35900	KRT20
0,004	-1,3	phenol sulfotransferase 1A5/1A possible alternative splicing form	P50224	SULT1A3
0,007	-1,31	Inorganic pyrophosphatase	Q15181	PPA1
0,025	-1,31	Thiopurine S-methyltransferase	P51580	TPMT
8,44E-05	-1,32	Enoyl-CoA hydratase, mitochondrial	P30084	ECHS1
0,004	-1,33	Protein DJ-1 (Oncogene DJ1)	Q99497	PARK7
0,045	-1,34	LGALS4 protein	Q6FHZ4	LGALS4
0,044	-1,35	Abhydrolase domain-containing protein 14B (22 kDa protein)	Q96IU4	ABHD14B
0,03	-1,35	LIM and SH3 domain protein 1	Q14847	LASP1
0,032	-1,35	3-mercaptopyruvate sulfurtransferase	P25325	MPST
0,002	-1,35	Phosphoglycerate mutase 1 (Brain) variant	Q53G35	PGAM1
0,007	-1,35	Thioredoxin-dependent peroxide reductase, mitochondrial	P30048	PRDX3
0,003	-1,37	Apoptosis-associated speck-like protein containing a CARD	Q9ULZ3	PYCARD
0,012	-1,38	Peptydil-prolyli cis-trans isomerase	P62937	PPIA
0,006	-1,43	Pyridoxine-5'-phosphate oxidase	Q9NVS9	PNPO
0,005	-1,43	Isoform short of 14-3-3 protein beta/alpha	A4K2U9	YWHAB
0,008	-1,45	22 kDa Protein (tyrosine 3-monooxygenase/tryptophan 5-monooxygenase activation protein, epsilon polypeptide [synthetic construct]) (14-3-3 epsilon)	P62258	YWHAE

0,001	-1,46	Nucleoside diphosphate kinase A	P15531	NME1
0,007	-1,46	Peptidyl-prolyl cis-trans isomerase A	P62938	PPIA
0,019	-1,46	Superoxide dismutase (Mn), mitochondrial precursor	P04179	SOD2
0,005	-1,47	Alcohol dehydrogenase [NADP+]	P14550	AKR1A1
0,025	-1,47	Fatty acid-binding protein, liver	P07148	FABP1
0,001	-1,5	Proteasome subunit beta type 2	P49721	PSMB2
0,013	-1,51	Myosin regulatory light chain 2, nonsarcomeric	P19105	MRCL3
0,014	-1,51	Phosphatidylethanolamine-binding protein 1	P30086	PEBP1
0,001	-1,52	Cathepsin D	P07339	CTSD
0,003	-1,52	EF-hand domain-containing protein D2	Q96C19	EFHD2
0,017	-1,53	Prohibitin	P35232	PHB
0,008	-1,54	Annexin A1	P04083	ANXA1
0,013	-1,55	Alpha-soluble NSF attachment protein	P54920	NAPA
0,002	-1,57	14-3-3 protein zeta/delta	P63104	YWHAZ
0,009	-1,59	Cofilin-1	P23528	CFL1
0,002	-1,59	NADH dehydrogenase (ubiquinone) flavoprotein 2, mitohondrial precursor	P19404	NDUFV2
5,43E-04	-1,6	Eukaryotic translation initiation factor 5A-1	P63241	EIF5A
0,038	-1,6	Bile salt sulfotransferase	Q06520	SULT2A1
0,012	-1,6	Transthyretin	P02766	TTR
0,004	-1,61	Cytochrome b5	P00167	CYB5A
0,006	-1,64	Glutathione S-transferase theta-1	P30711	GSTT1
0,001	-1,68	Annexin A5	P08758	ANXA5
0,008	-1,69	cytidylate kinase (UMP-CMP kinase)	P30085	CMPK1
0,011	-1,71	Actin	P62736	ACTA
0,001	-1,83	proteasome activator subunit 1 isoform 1 (29KDa protein)	Q06323	PSME1
0,006	-1,86	Endoplasmic reticulum protein ERp29 precursor	P30040	ERP29

Suplementary table 3. Clinical Correlations. Spearman's correlations among relative protein expression and clinical variables were performed. Both high correlations ($r_s > 0.55$) and low P (close to P = 0.05) are represented in bold. P-values <0.05 are highlighted in yellow.

	Severity of abdominal pain		Frequency of abdominal pain		Stool frequency		Stool consistency	
	r_s	P	r_s	P	r_s	P	r_s	P
pCFL1/CFL1	0.1458	0.6689	0.07909	0.8172	-0.1975	0.4176	-0.2095	0.3620
pCFL1/CFL1 M	0.1449	0.7841	-0.4708	0.3460	0.1380	0.7038	0.06791	0.8521
pCFL1/CFL1 F	0.1000	0.9729	0.5270	0.3615	**-0.6588**	**0.0536**	-0.3486	0.2934
pCFL1/ACTB	-0.1230	0.7186	0.1675	0.6226	-0.4004	0.0893	-0.3393	0.1324
pCFL1/ACTB M	0.2609	0.6175	-0.4708	0.3460	-0.05646	0.8769	0.08643	0.8123
pCFL1/ACTB F	0.2000	0.7411	0.5270	0.3615	**-0.8696**	**0.0023**	-0.3349	0.3141
TESK-1/ACTB	-0.1048	0.7591	-0.01861	0.9567	-0.1600	0.5109	-0.3090	0.1729
TESK-1/ACTB M	-0.1160	0.8268	-0.2942	0.5714	0.3639	0.3013	0.2593	0.4694
TESK-1/ACTB F	0.1000	0.8729	0.000	1.0000	**-0.7379**	**0.0232**	**-0.6009**	**0.0505**
VCL/ACTB	0.2141	0.5272	0.2140	0.5272	-0.2869	0.2337	-0.2253	0.3261
VCL/ACTB M	0.05798	0.9131	-0.05885	0.9118	0.1279	0.7561	0.2778	0.4370
VCL/ACTB F	**0.900**	**0.0374**	**0.7906**	**0.1114**	**-0.7379**	**0.0232**	**-0.5826**	**0.0600**

Supplementary figure 4. Full length blot for the western blots from figure 4. Full length western blot (left: image of developed blot; right: image of the membrane) are represented indicating antibody dilution, exposure time, molecular weight of the expected band, cropped region and an unrelated sample used as internal control marked with asterisk. A) phosphorylated cofilin 1, B) Total Cofilin 1, C) TESK1 and D) Actin from left western blot fig 2 supp; E) phosphorylated cofilin 1, F) Total Cofilin 1, G) TESK1 and H) Actin from central western blot fig 2 supp; I) phosphorylated cofilin 1, J) Total Cofilin 1, K) TESK1 and L) Actin from rigth western blot fig 2 supp and western blot from fig 4a.

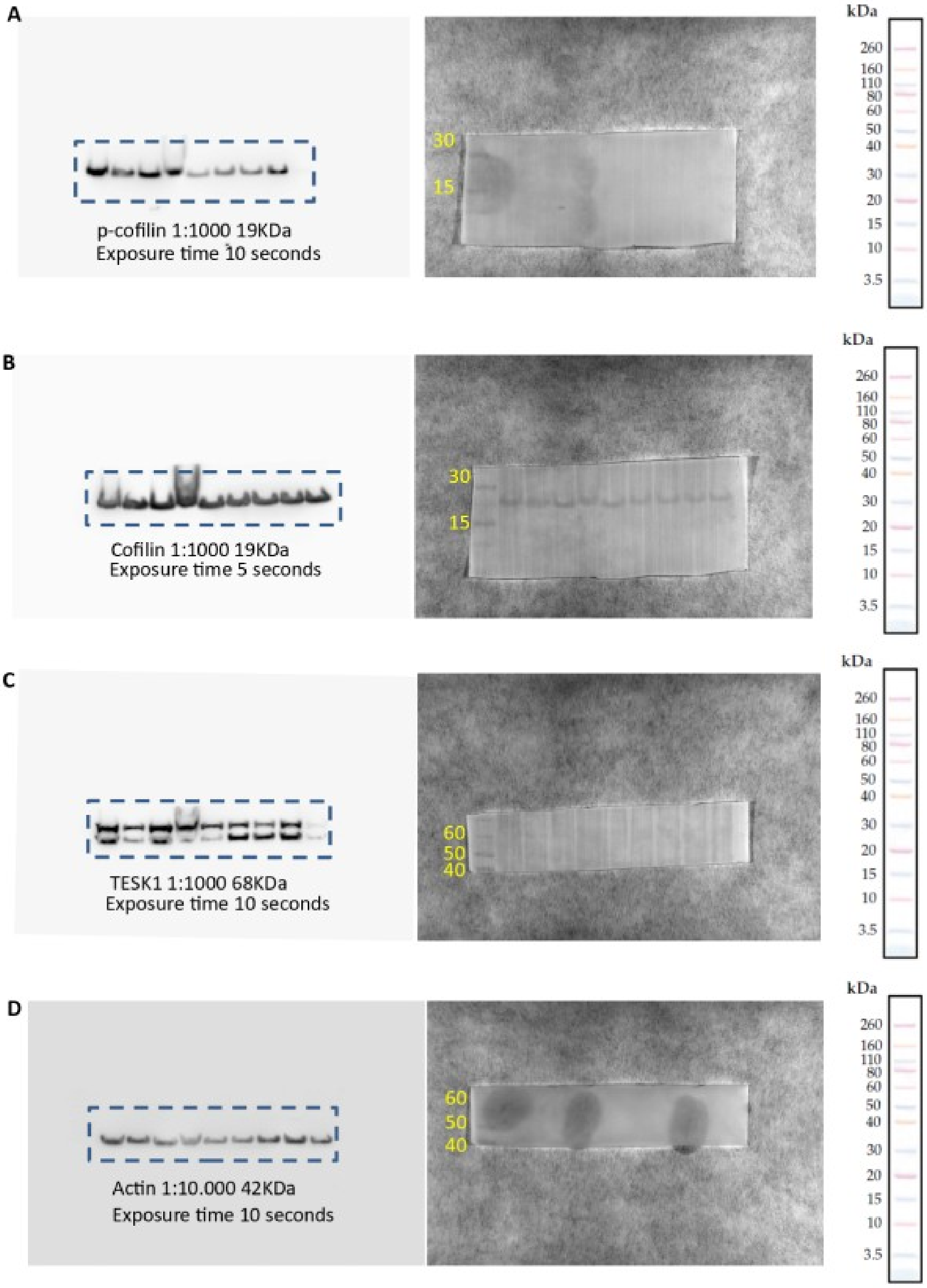
A
p-cofilin 1:1000 19KDa
Exposure time 10 seconds
30
15
B
Cofilin 1:1000 19KDa
Exposure time 5 seconds
30
15
C
TESK1 1:1000 68KDa
Exposure time 10 seconds
60
50
40
D
Actin 1:10.000 42KDa
Exposure time 10 seconds
60
50
40
kDa
260
160
110
80
60
50
40
30
20
15
10
3.5

E

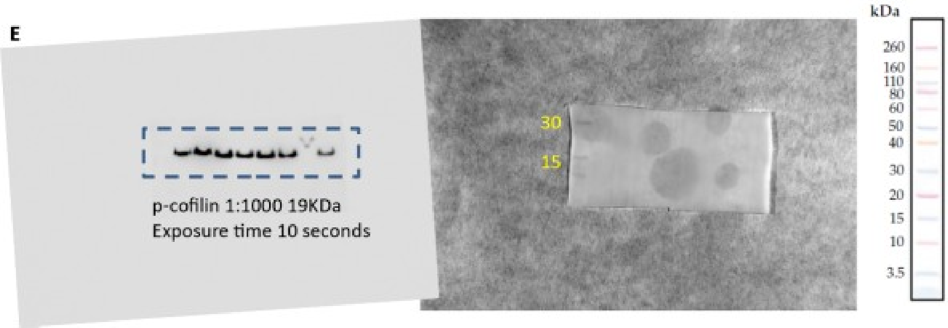

p-cofilin 1:1000 19KDa
Exposure time 10 seconds
30
15
kDa
260
160
110
80
60
50
40
30
20
15
10
3.5

F

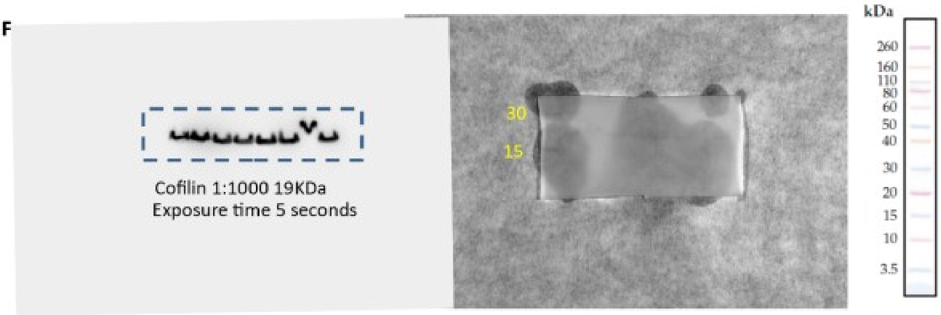

Cofilin 1:1000 19KDa
Exposure time 5 seconds
30
15
kDa
260
160
110
80
60
50
40
30
20
15
10
3.5

G

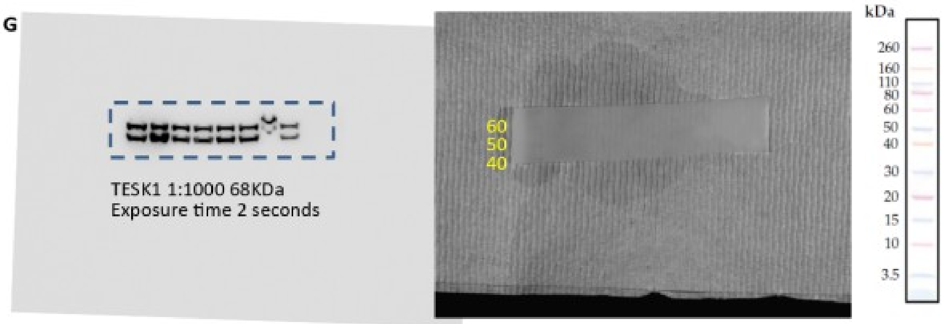

TESK1 1:1000 68KDa
Exposure time 2 seconds
60
50
40
kDa
260
160
110
80
60
50
40
30
20
15
10
3.5

H

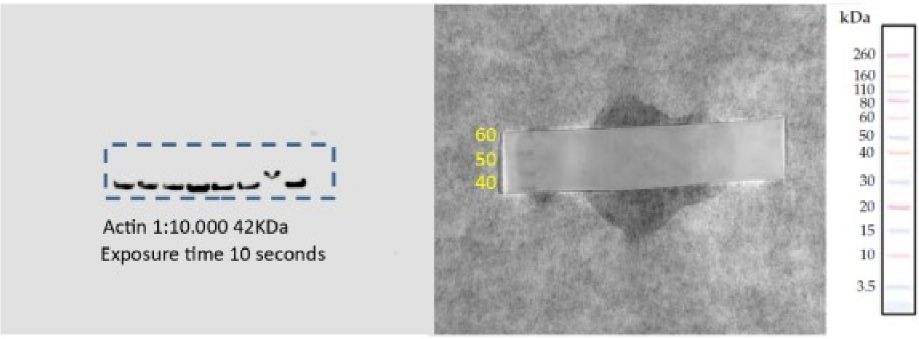

Actin 1:10.000 42KDa
Exposure time 10 seconds
60
50
40
kDa
260
160
110
80
60
50
40
30
20
15
10
3.5

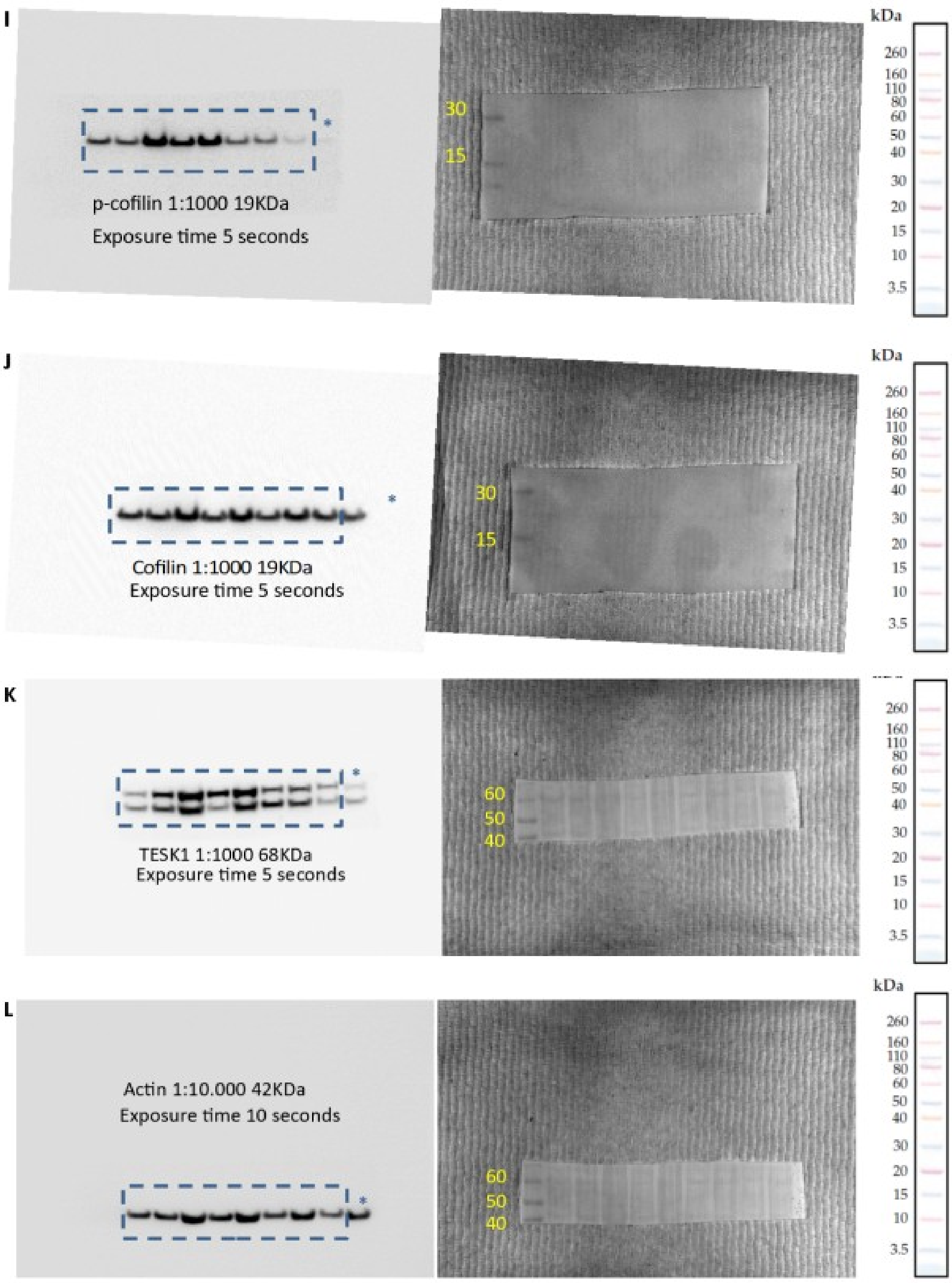
I
*
p-cofilin 1:1000 19KDa
Exposure time 5 seconds
30
15
kDa
260
160
110
80
60
50
40
30
20
15
10
3.5
J
*
Cofilin 1:1000 19KDa
Exposure time 5 seconds
30
15
kDa
260
160
110
80
60
50
40
30
20
15
10
3.5
K
*
TESK1 1:1000 68KDa
Exposure time 5 seconds
60
50
40
260
160
110
80
60
50
40
30
20
15
10
3.5
L
Actin 1:10.000 42KDa
Exposure time 10 seconds
*
60
50
40
kDa
260
160
110
80
60
50
40
30
20
15
10
3.5
